# The Effect of Attentional Cueing and Spatial Uncertainty in Visual Field Testing

**DOI:** 10.1371/journal.pone.0150922

**Published:** 2016-03-03

**Authors:** Jack Phu, Michael Kalloniatis, Sieu K. Khuu

**Affiliations:** 1 Centre for Eye Health, University of New South Wales, Kensington, New South Wales, Australia; 2 School of Optometry and Vision Science, University of New South Wales, Kensington, New South Wales, Australia; University of Waterloo, CANADA

## Abstract

**Purpose:**

To determine the effect of reducing spatial uncertainty by attentional cueing on contrast sensitivity at a range of spatial locations and with different stimulus sizes.

**Methods:**

Six observers underwent perimetric testing with the Humphrey Visual Field Analyzer (HFA) full threshold paradigm, and the output thresholds were compared to conditions where stimulus location was verbally cued to the observer. We varied the number of points cued, the eccentric and spatial location, and stimulus size (Goldmann size I, III and V). Subsequently, four observers underwent laboratory-based psychophysical testing on a custom computer program using Method of Constant Stimuli to determine the frequency-of-seeing (FOS) curves with similar variables.

**Results:**

We found that attentional cueing increased contrast sensitivity when measured using the HFA. We report a difference of approximately 2 dB with size I at peripheral and mid-peripheral testing locations. For size III, cueing had a greater effect for points presented in the periphery than in the mid-periphery. There was an exponential decay of the effect of cueing with increasing number of elements cued. Cueing a size V stimulus led to no change. FOS curves generated from laboratory-based psychophysical testing confirmed an increase in contrast detection sensitivity under the same conditions. We found that the FOS curve steepened when spatial uncertainty was reduced.

**Conclusion:**

We show that attentional cueing increases contrast sensitivity when using a size I or size III test stimulus on the HFA when up to 8 points are cued but not when a size V stimulus is cued. We show that this cueing also alters the slope of the FOS curve. This suggests that at least 8 points should be used to minimise potential attentional factors that may affect measurement of contrast sensitivity in the visual field.

## Introduction

Contrast, which can be defined as the difference in luminance of an object relative to its background, provides information regarding the visual scene, which can range from fine detail, such as reading words on a page, to navigation in the world [1]. Contrast detection begins when light is absorbed at the photoreceptor before being processed by retinal elements and further up in cortical regions by neuronal channels [2–6]. In eye disease such as glaucoma loss of detector units (e.g. retinal ganglion cells) may result in deficits in contrast detection at different locations in the visual field [7, 8], and as such, assessment of this ability is useful in the diagnosis and management of ocular disease.

One commonly used technique in both laboratory and clinical settings to assess contrast sensitivity in the visual field is white-on-white standard automated perimetry (SAP) in which a Goldmann size III stimulus is presented at multiple locations for a brief, constant duration of 200 ms [9]. The advantages of SAP over methods such as confrontation visual fields or kinetic perimetry is that contrast detection can be measured at many discrete and predetermined points, and hence visual behaviour and performance at multiple locations across the visual field can be immediately quantified [9–11].

In SAP, contrast sensitivity is measured by sequentially presenting and testing stimuli at pseudo-random locations in the visual field, and observers are required to subjectively respond to when they detect the stimulus. Using this paradigm, it is almost impossible for the observer to predict where the stimulus will appear [12, 13]. Accordingly, conventional SAP testing procedures are likely to be affected by *spatial uncertainty*.

Spatial uncertainty is an *extrinsic* factor that is inherent to the testing and affects *intrinsic* uncertainty and the way in which the observer responds to a stimulus [14]. Spatial uncertainty can be defined as uncertainty arising from the observer having to allocate visual attention only to specific regions of the entire visual field, whilst objects appear in other, unattended regions of the field. However, the limited capacity of spatial attention might mean that only specific regions of the visual field are attended for processing [15, 16]. This is problematic for visual field testing as randomly presented elements introduce spatial uncertainty, which in turn affects the detectability of targets, especially at contrast levels close to threshold [17, 18]. The degree to which spatial uncertainty affects visual field testing has yet to be systematically determined.

In visual field testing observers might miss a target on a particular trial because of inattention and not because it cannot be detected. Accordingly, to overcome spatial uncertainty, the stimulus contrast must be higher than the threshold limit for detection, and this has been shown in a number of laboratory-based studies [14, 15, 19–21]. These studies also used cues, such as visual markers at the spatial location prior to the appearance of the stimulus, to overcome spatial uncertainty, and this was shown to increase contrast sensitivity at those locations. More recently, Khuu and Kalloniatis [22] used testing procedures analogous to SAP to show that spatial uncertainty affects both stimulus detectability and observer criterion bias. Importantly, these factors contribute majorly to contrast detection performance, and ultimately the threshold value that is reported by SAP instruments.

There are situations in SAP where spatial uncertainty may be reduced. First, some perimeters have the option for retesting individual points, which is clinically useful when singular isolated points appear suspicious. The effect of retesting individual points where spatial uncertainty is minimised (due to their presentation at a non-random location, which may be predictable) on the measured threshold is not known. Second, custom test paradigms may use fewer test points than that of SAP, which may result in less spatial uncertainty, and therefore might not be immediately comparable to SAP results. Although there are no visual cues available on commercial SAP instruments, it is possible for observers to anticipate or to be verbally cued the locations of subsequent stimuli. Therefore, while SAP is the gold standard for clinical assessment of the visual field, there is considerable interest in addressing factors which may affect its ability to detect altered visual function in diseased states.

The aim of the present study was to systematically investigate the potential influence of spatial uncertainty on contrast sensitivity through verbal cueing. We compared performance between conditions in which the spatial location of targets presented in the Humphrey Visual Field Analyzer (HFA) are uncued or cued by providing the observer with prior knowledge regarding the number of points to be tested and their spatial location through verbal cueing. We obtained thresholds at 8 different meridians, for stimuli of Goldmann sizes I, III and V, and with 1, 2, 4 and 8 points, and compared this to an uncued condition, when using the 30–2 full threshold paradigm on the HFA. We expect that size would have some effect on spatial uncertainty, as it has been shown to affect measurements of contrast sensitivity at different locations in the visual field [11, 22–25]. The clinical-based testing gives information about the absolute difference in sensitivity, but cannot determine the frequency-of-seeing (FOS) curves, which provide information about changes in threshold, and can also act as a surrogate measure for certainty with its shape and slope parameter [26, 27]. Thus, we subsequently measured the psychometric functions for a subset of observers across similar conditions, hypothesising that spatial uncertainty in detecting a smaller test size is greater than when using a larger target, particularly at peripheral locations at which detectability is already reduced [28, 29].

## Methods

### Participants

Six observers (3 male, 3 female; mean age: 35.8 years) participated in the clinical-based testing phase. Two were authors of the study (JP and MK), and the four others were experienced psychophysical observers, but were naïve to the aims of the study. Four of these observers (2 male, 2 female; mean age: 32.3 years) underwent further laboratory-based psychophysical testing in the second phase. One of these observers designed the experiment (JP) and the other three observers were naïve to the purpose of the tests. All observers had substantial previous experience undergoing clinical perimetric testing, which has been shown to be affected by practice effects [30]. All had normal or corrected to normal visual acuity (range of refractive error: +1.00D to -4.37D equivalent sphere). All observers had undergone ocular examination that included fundus examination, optical coherence imaging, and tonometry, which found no signs of ocular disease. Additionally, all observers gave their written informed consent prior to participating in the present study. Ethics approval was given by the Institutional Review Board of University of New South Wales Ethics committee, and the experiment followed the tenets of the Declaration of Helsinki. Testing was performed with one eye (the other eye was patched) with natural pupils. The order of testing of each of the sizes was randomised to minimise order effects. These data were collected over a number of testing sessions to reduce the effects of fatigue.

### Stimulus and Apparatus—HFA-Based Testing

We used the HFA to measure contrast sensitivity at 77 spatial locations (including the fovea) of the 30–2 testing pattern using the full threshold paradigm and Goldmann sizes I, III and V. Age and refractive error appropriate trial lenses were used throughout the testing process. The full threshold paradigm was chosen as other different thresholding paradigms, such as suprathreshold or SITA, result in altered threshold levels [31, 32]. Thresholds were measured at least three times for each observer using the full threshold paradigm, and these values were averaged to provide an estimate of the contrast detection threshold at each location.

The 30–2 full threshold test results were used as the baseline reference, as we were interested in examining the role of spatial uncertainty in contrast sensitivity measured with this test, which is commonly used in clinical assessments. This was also referred to as the uncued condition, as the 76 points of the 30–2 test pattern (the fovea may be considered inherently cued, as attention is specifically directed to its location) are presented in a pseudo-random order at different spatial locations, beginning with four seeding points. Hence, spatial uncertainty is maximal for this pattern when all points are tested.

### HFA Cueing Paradigm

The Custom Test function of the HFA, which allows a custom test pattern to be programmed using Cartesian coordinates (in degrees) in the central 30-degree visual field, was utilised to generate stimuli for the cued portion of the test where the location and the size of the stimuli were varied. Testing was conducted by cueing 1, 2, 4 or 8 points at various spatial locations. The observers were verbally informed of the number of points that would appear and their respective spatial locations prior to each testing condition. For example, they might be told that: “One point will appear up and slightly to your right, at your 1 o’clock orientation” for a 1-point cued testing condition. These cueing conditions were tested in a random order. An initial pilot study was conducted that included 12 and 16 points cued. It was found that the effect of spatial cueing was minimal by approximately 8 points, i.e. no effect of spatial cueing when more than 8 points were cued. Therefore, participants were tested up to 8 points cued. However, if there was no plateau effect found when 8 points were cued, conditions of 12 points or more cued were conducted until a plateau effect was found, i.e. no difference was found from when 76 points were tested using the complete 30–2 test pattern described above.

**Fig 1:**
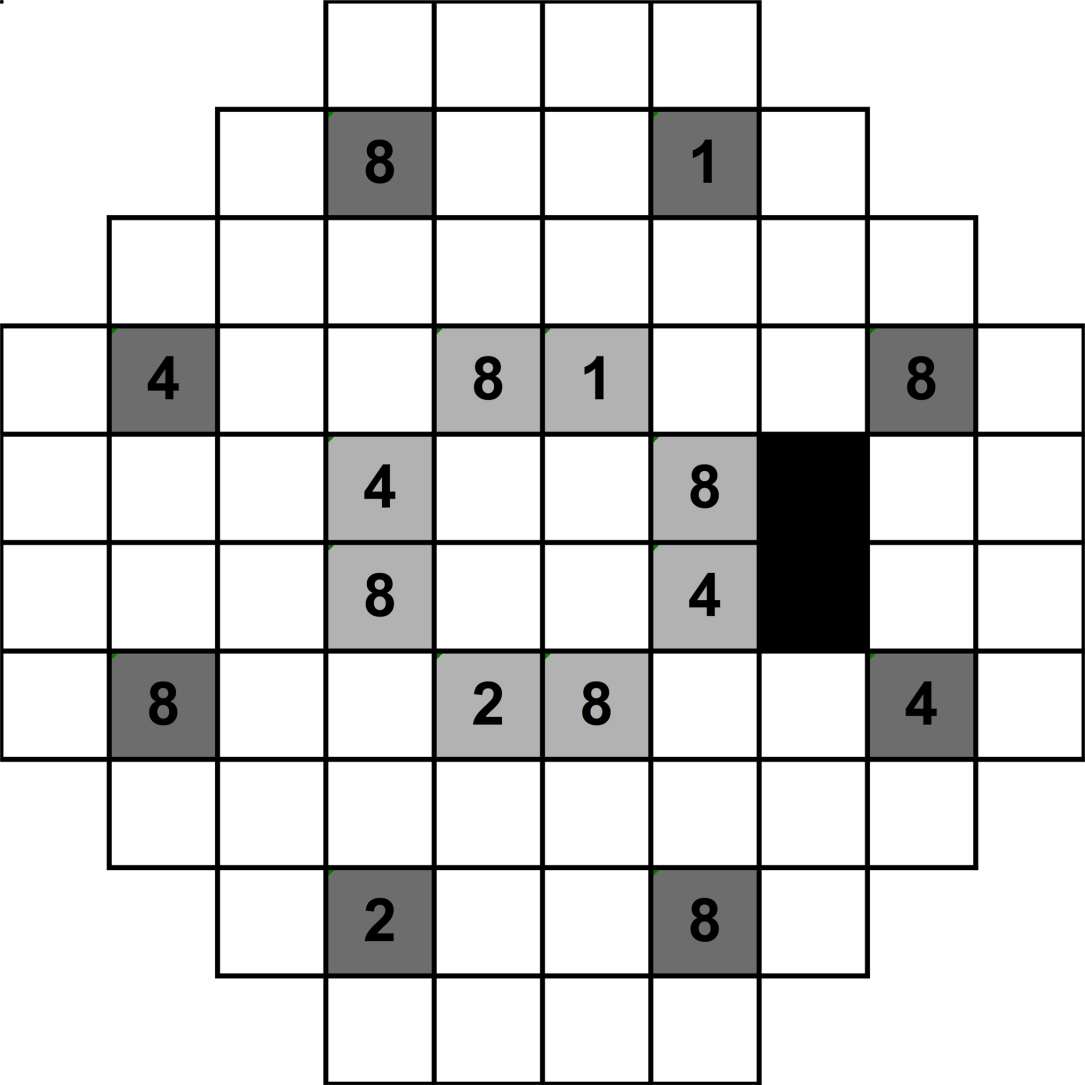
A schematic of the HFA 30–2 testing pattern. This is representative of the right eye examination, where the black boxes represent the area of the blind spot. There are 8 possible locations for the peripheral (dark grey boxes) and mid-peripheral (light grey boxes) conditions. The numbers depict examples of how the points are cued. For example, when 1 point is cued, then square 1 is cued; when 2 points are cued, then squares 1 and 2 are used; when 4 points are cued, then squares 1, 2 and 4 are used; when 8 points are cued, then squares 1, 2, 4 and 8 are used. For example, using this schematic, when one point was cued in the “peripheral” condition, the observer would be told: “One point will appear up and slightly to your right, at approximately 1 o’clock, in the periphery”. These spatial locations were rotated and varied so that each of the locations was presented during different cueing sessions.

The abovementioned procedures were tested using Goldmann size I, III and V stimuli, as previous studies have shown that less variability in thresholds measured using larger stimuli compared to smaller stimuli [27, 33, 34]. This is hypothesised to be due to differences in spatial certainty [27, 33, 34]. Thus, each observer had a total of at least nine full threshold visual field results (at least three per size). Test points chosen for the cueing procedure were at two eccentric locations: approximately 9.5° and 22.8° from fixation, as shown in Fig 1, which we refer to in the text as “mid-peripheral” and “peripheral” locations respectively, for simplicity. The mid-peripheral and peripheral locations were tested separately. The peripheral most points of the 30–2 test pattern have been shown to display the greatest variability [35], and may be affected by lens rim artefacts [36]. Therefore, the second outermost ring of points was selected, as these are also utilised in the 24–2 paradigm, typical of clinical practice. These two eccentricities were chosen to study the effects of spatial uncertainty and distance from fixation on contrast sensitivity. The Cartesian coordinates in degrees for these points are as follows: (-9,+21), (+9,+21), (-21,+9), (+21,+9), (-21,-9), (+21,-9), (-9,-21) and (+9,-21). The mid-peripheral points tested were along the same meridian as that of the peripheral points, with Cartesian coordinates: (-3,+9), (+3,+9), (-9,+3), (+9,+3), (-9,-3), (+9,-3), (-3,-9) and (+3,-9). When cueing these points, combinations were chosen to maintain substantial difference between them, rather than having them proximal to each other. These patterns were rotationally symmetrical about fixation. For example, a 2 points cued condition could include a combination of (-3,+9) and (+3,-9), which included points directly opposite to each other. Fig 1 also shows possible combinations of points cued: one possible combination of 2 points cued is marked by “1” and “2”, and one combination of 4 points cued in this Figure occurs at locations marked by “1”, “2” and “4”. A rotationally symmetrical combination of 2 points cued in this Figure would at locations marked “4”, for example.

We measured at least twenty-five thresholds for each cued condition (e.g. 1-point cued had twenty-five thresholds in total amongst all locations tested), with at least three thresholds obtained at each test location. Reliability indices, in-built gaze tracking on the HFA, and external monitoring of fixation via the instrument’s camera were also monitored. Observers had false positive, false negative and fixation loss rate of less than 5% in both baseline testing and combined cued testing, with no difference found between uncued and cued conditions, consistent with the work of Shaw [37], who showed that attending to multiple sources of information improves sensitivity without significantly altering the false alarm rate.

### Stimulus and Apparatus–Laboratory-Based Testing

**Fig 2:**
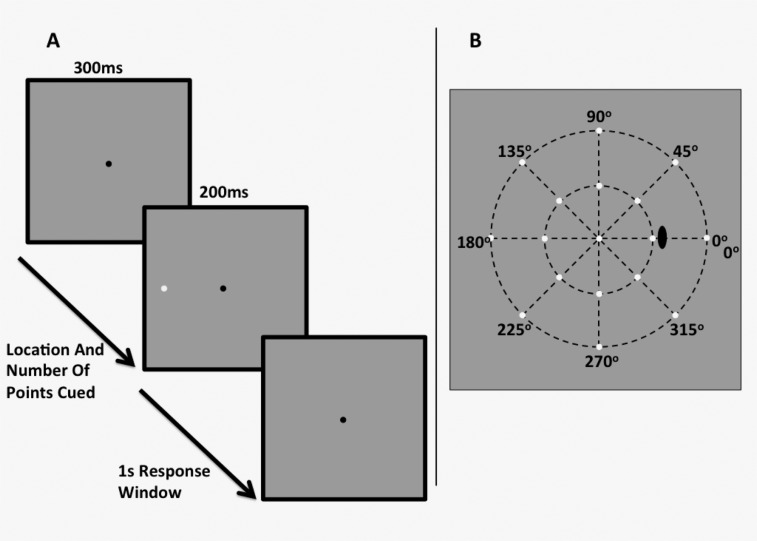
A schematic of the experimental stimulus and example procedure when one point to the observer’s left hand side is cued (A), and the stimulus locations presented in the “mid-periphery” 12.7° (inner ring) and “periphery” 29.7° (outer ring) conditions.

Stimuli were white circular spots of light presented on a white-gray background (10 cd/m^2^) for 200 ms (see: Fig 2A and 2B). We used three stimulus sizes, equivalent to that of the Goldmann sizes I, III and V on the HFA (0.11°, 0.43° and 1.73° in diameter respectively). These stimuli were presented at meridians (from right horizontal in clockwise fashion) of 0°, 45°, 90°, 135°, 180°, 225°, 270° and 315°, and at eccentricities of 12.7° (Fig 2A) and 29.7° (Fig 2B). These locations were slightly more eccentric compared to that of Experiment 1, as we were not limited by the grid-like pattern of the HFA. This allowed for the determination of whether the effect persists at different eccentricities, particularly farther in the periphery, hypothesising that if the effect of cueing is apparent and robust, it will also appear at different eccentricities, and that such an effect is not instrument-specific. A black fixation mark (0.06° x 0.06°, Weber Contrast -0.2) was placed at the centre of the screen, upon which the participant was instructed to fixate during the trial. Stimuli were generated on an iMac computer using custom written software in MATLAB (Mathworks, version 7), and were presented on the iMac monitor driven at a frame rate of 60 Hz. A head and chin rest was used to ensure a constant viewing distance. These observers exhibited good reliability during the testing phase using the HFA (with fixation loss, false positive and false negative rates of <5%), and so fixation was monitored externally by the examiner. For the 12.7° test eccentricity, we used a viewing distance of 30 cm. Due to the technical limitations of having a flat-screen monitor instead of a projection system as in the HFA, we had to halve the working distance to 15 cm for the 29.7° eccentricity. To mitigate the optical effects of trial lens, all subjects wore contact lenses to correct for refractive error and working distance.

### Procedure

Participants were instructed to maintain central fixation, and after a period of 300 ms, the presentations began. The stimuli were presented for 200 ms, after which the background was shown for up to 1 s, during which the subject could respond. If a response was entered, the next stimulus was presented; if not, then after 1 s, the next stimulus was shown. There was no auditory cue to signal the onset of stimulus presentation, to replicate the conditions of the HFA. We cued the number of points that would appear and their location in each trial, for example: “one point at the left-hand side”; or “four points appearing up, down, left and right”. We cued 1, 2, 4 or 8 points, since we had found that using the HFA there was almost no effect of attentional cueing on contrast sensitivity when 8 points were cued. The task of the observers was to indicate whether they saw a stimulus by pressing a button on a computer keyboard. They did not have to indicate if they did not see the spot.

We utilised Method of Constant Stimuli (MoCS), presenting stimuli at, at minimum, nine possible contrast levels for each combination of stimulus size and eccentricity. Due to the length of time required to measure the FOS curve for each location, we only measured the responses obtained from one stimulus location: the nasal (i.e. left horizontal, or 180° condition) location. Hence, within each block of trials, the nine contrast levels of the nasal location were presented 10 times for a total of 90 trials. At each other test location for conditions involving more than one point cued, we randomly presented trials there to maintain division of attention. Since we did not measure the FOS curves at these locations, we presented the stimuli at higher contrast levels, as attention would not be adequately captured if approximately half of the trials were below threshold at those locations, which MoCS assumes [38]. We converted the output dB value obtained from the HFA in the clinical testing phase to Weber contrast levels to obtain an approximate starting threshold contrast level individually for each subject, as there were individual variations in contrast sensitivity with size and location. From this contrast level, we chose at least four contrast levels above and at least four contrast levels below to present, modulating it according to stimulus size. During the pilot phase of testing, we further refined the contrast levels obtained for each subject, such that the contrast level initially converted from the results of the HFA were slightly different in the psychophysical experiment. We also found that under some conditions, more than four contrast levels above or below were required to reach a plateau, and increased the number of contrast levels accordingly (S1 Table).

### Statistical Analysis

For the clinical-based testing phase, we determined the difference in the thresholds between cued and its uncued (“baseline”) conditions at each spatial location. A Kolmogorov-Smirnov test with Dallal-Wilkinson-Lilliefor P value found that the differences in threshold for each cueing condition (e.g. 1 point cued, 2 points cued) were normally distributed. We averaged the differences across all spatial locations for each cued condition and plotted the difference in threshold (y) as a function of number of points cued (x). Positive values along the y-axis indicate that the cued condition lead to greater contrast sensitivity (i.e., lower contrast detection thresholds) relative to the uncued baseline conditions. A y-value of 0 indicates there is no difference between cued and baseline. The error bars in these graphs represent 1 Standard Error of the Mean. A one-phase decay nonlinear regression curve (of the form y = Y_0_.e^(-K.x)^, where Y_0_ represents the y-intercept of the fitted curve, i.e. when x = 0, and K is the rate constant) was fitted to this data with the plateau constrained to asymptote towards 0.0 (GraphPad Prism version 6).

For the laboratory-based testing phase, we plotted the proportion seen as a function of contrast (log Weber contrast Δ*L*/*L*) for each of the six combinations of stimulus size and location, within which each number of points cued condition was included. We fitted FOS curves using a sigmoidal nonlinear regression curve with variable slope (GraphPad Prism version 6). We extracted the EC50 and the slope, since using MoCS paradigm utilised a threshold frequency at 50% seen; hence EC50 represented the threshold value in our experiment. We did not combine individual data for analysis, except where absolute differences were used, for which we fitted one-phase decay nonlinear regression curves.

## Results

### HFA-Based Testing

**Fig 3:**
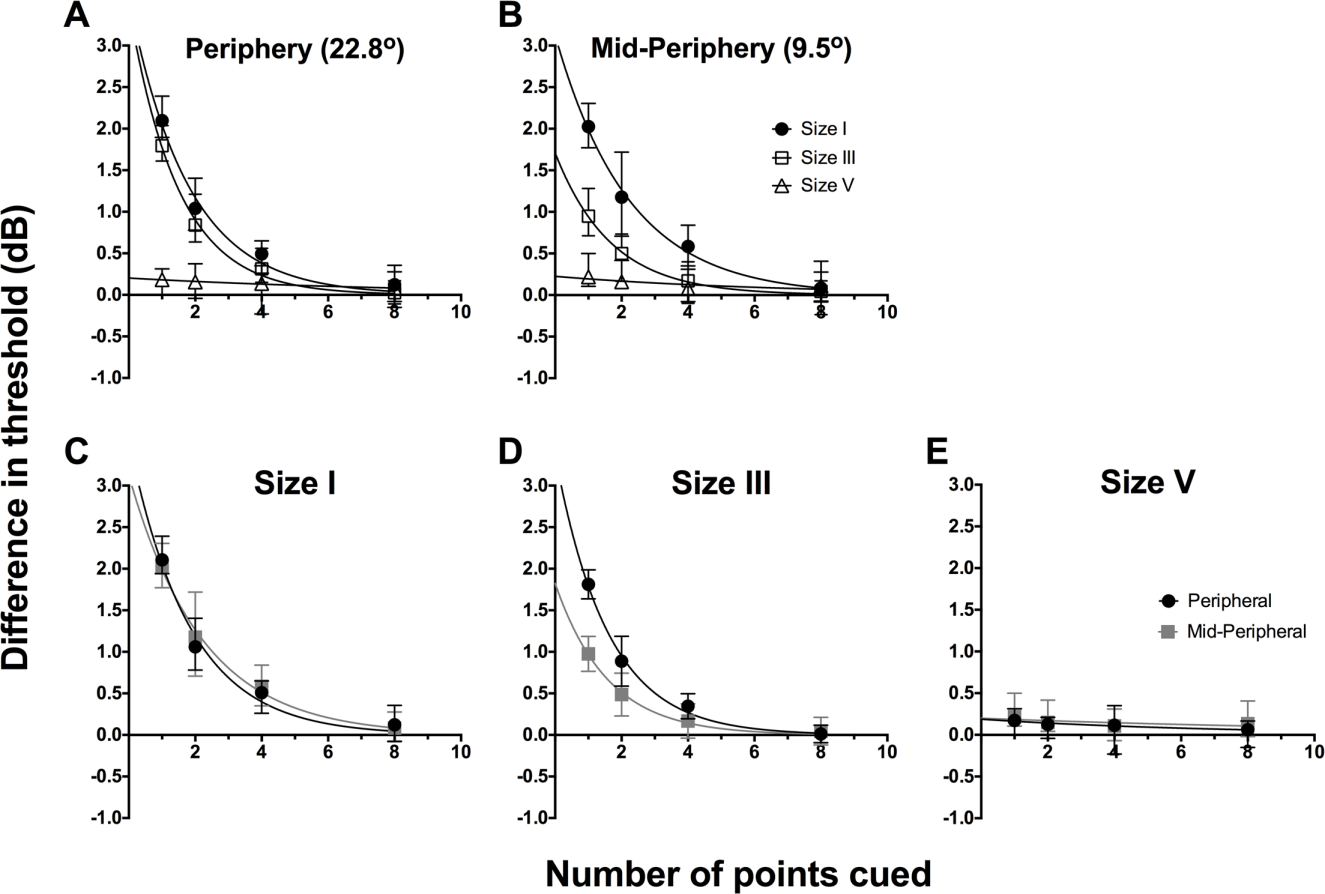
Combined data (n = 6) for the difference in threshold between cued and uncued conditions (dB) as a function of the number of points cued. The same data set was plotted in two ways: by stimulus location (A, B) and by stimulus size (C-E). For all graphs, solid lines represent the best-fit one-phase nonlinear regression through those points, and error bars represent the range. The top row represents the functions sorted by peripheral (A) and mid-peripheral (B) stimulus location, and within each graph, we present size I (black circles), size III (open squares) and size V (open triangles) results separately. The bottom row represents the functions sorted by stimulus size: size I (C), size III (D) and size V (E), and within each graph, we present peripheral (black circles) and mid-peripheral (grey squares) separately. The resultant equations for the peripheral conditions were: size I, y = 3.574e^-0.558x^; size III, y = 3.482e^-0.675x^; and size V, y = -0.014x + 0.172 (linear regression was utilised for size V). The resultant equations for the mid-peripheral conditions were: size I, y = 3.124e^-0.452x^; size III, y = 1.709e^-0.570x^; and size V, y = -0.009x + 0.187 (linear regression was utilised for size V).

To examine the effect of cueing, we first determined the difference in threshold between the particular points tested in the cued conditions and the corresponding points in the uncued condition. Then, these threshold differences were separately averaged for different stimulus sizes and spatial locations. We plot the combined data for all 6 observers for the difference in threshold between cued and uncued conditions as a function of number of points cued in Fig 3 (individual data are shown in S1 and S2 Figs). We first show the average threshold difference for different test sizes (different symbols) for peripheral (Fig 3A) and mid-peripheral testing locations (Fig 3B). Repeated-measures two-way ANOVA was conducted for the combined data set from all six observers to examine the effect of number of points cued (factor 1, four levels) and stimulus size (factor 2, three levels). In the peripheral location, there was a significant effect of stimulus size (F(2,40) = 237.2, *p*<0.0001), number of points cued (F(3,20) = 91.37), *p*<0.0001), and significant interaction (F(6, 40) = 69.66, *p*<0.0001). There were similar significant findings in the mid-periphery, for the effects of size (F(2,40) = 99.82, *p*<0.0001) and number of points cued (F(3,20) = 106.6, *p*<0.0001). There was also a significant interaction effect (F(6,40) = 21.62, *p*<0.0001), which indicated that the effect of cueing was dependent on stimulus size.

The exponential functions in Fig 3 (see Fig 3 caption for best fit parameters) appeared to fit the data well when a size I stimulus was used (peripheral: R^2^ = 0.93; mid-peripheral: R^2^ = 0.91). The functions for size III showed good fit with the peripheral data (R^2^ = 0.94), but the fit was poorer for the mid-peripheral condition (R^2^ = 0.79). In these data, we found a relative increase in contrast sensitivity when one point was cued, decaying exponentially until 8 points were cued. The size V results did not follow an exponential decay, as there was essentially no effect of cueing on its thresholds. We attempted a linear regression fit for the data, which fit the data better, but showed an essentially straight line (peripheral: R^2^ = 0.91; mid-peripheral: R^2^ = 0.36).

Fig 3C–3E show the difference in threshold between cued and baseline as a function of number of points cued for peripheral and mid-peripheral testing, separated by stimulus size I, III and V. Repeated-measures two-way ANOVA was conducted for the combined data set from all six observers to examine the effect of number of points cued (factor 1, four levels) and location tested (factor 2, two levels) on contrast thresholds. This analysis confirmed that changing the number of points cued had a significant effect (F(3,20) = 140.3, *p*<0.0001) when testing with a size I stimulus, whereby testing with one point cued increased contrast sensitivity by over 2 dB relative to baseline. There was no significant effect due to location of testing using stimulus size I (F(1,20) = 0.09304, *p* = 0.7635), indicating that the effect of cueing was the same for both peripheral and mid-peripheral testing locations (Fig 3C). For stimulus size III, this analysis confirmed that there was a significant effect due to changing the number of points cued (F(3,20) = 109.4, *p*<0.0001) and changing the location of testing from peripheral to mid-peripheral (F(1,20) = 42.92, *p*<0.0001) (Fig 3D). These factors also demonstrated significant interaction (F(3,20) = 11.36, *p* = 0.0001), which shows that for stimulus size III, the effect of number of points cued is dependent on the location being tested. For stimulus size V, there was no significant effect due to number of points cued (F(3,20) = 2.711, *p* = 0.0723) or location of testing (F(1,20) = 1.174, p = 0.2914).

### Laboratory-Based Psychophysical Testing

The individual FOS curves are depicted in S3–S6 Figs, plotting proportion seen as a function of log contrast (in dB, Δ*L*/*L*, where Δ*L* is the difference between the luminance of the stimulus from the background *L*). The dB output from the HFA is a measure of attenuation, rather than luminance; hence the direction of effect is opposite, i.e. an increase in dB found with the HFA is indicative of increased sensitivity to a more attenuated stimulus, whilst in an increase in dB in laboratory-based testing represents a required increase in stimulus intensity for detection (thus, lesser sensitivity). The black curves represent one point cued, blue two points, yellow four points, and red eight points. The top row (A-C) for each Figure shows the results for the 12.7° eccentricity and the bottom row (D-F) for the 29.7° condition. For all four observers, there was a leftward shift of the black curve for size I stimuli at both eccentricities, relative to the other curves; similarly, there was a small leftward shift of the black curve for size III stimuli presented at the 29.7° eccentricity. For size III stimuli at the 12.7° eccentricity and for size V stimuli at both eccentricities, there was no apparent difference in the positions of the curves. There were also some visible differences in the relative shape of the curves: the size I curves appeared to be flatter overall in comparison to size III and V results. Notably, the size V results displayed relatively steep FOS curves across all conditions.

**Fig 4:**
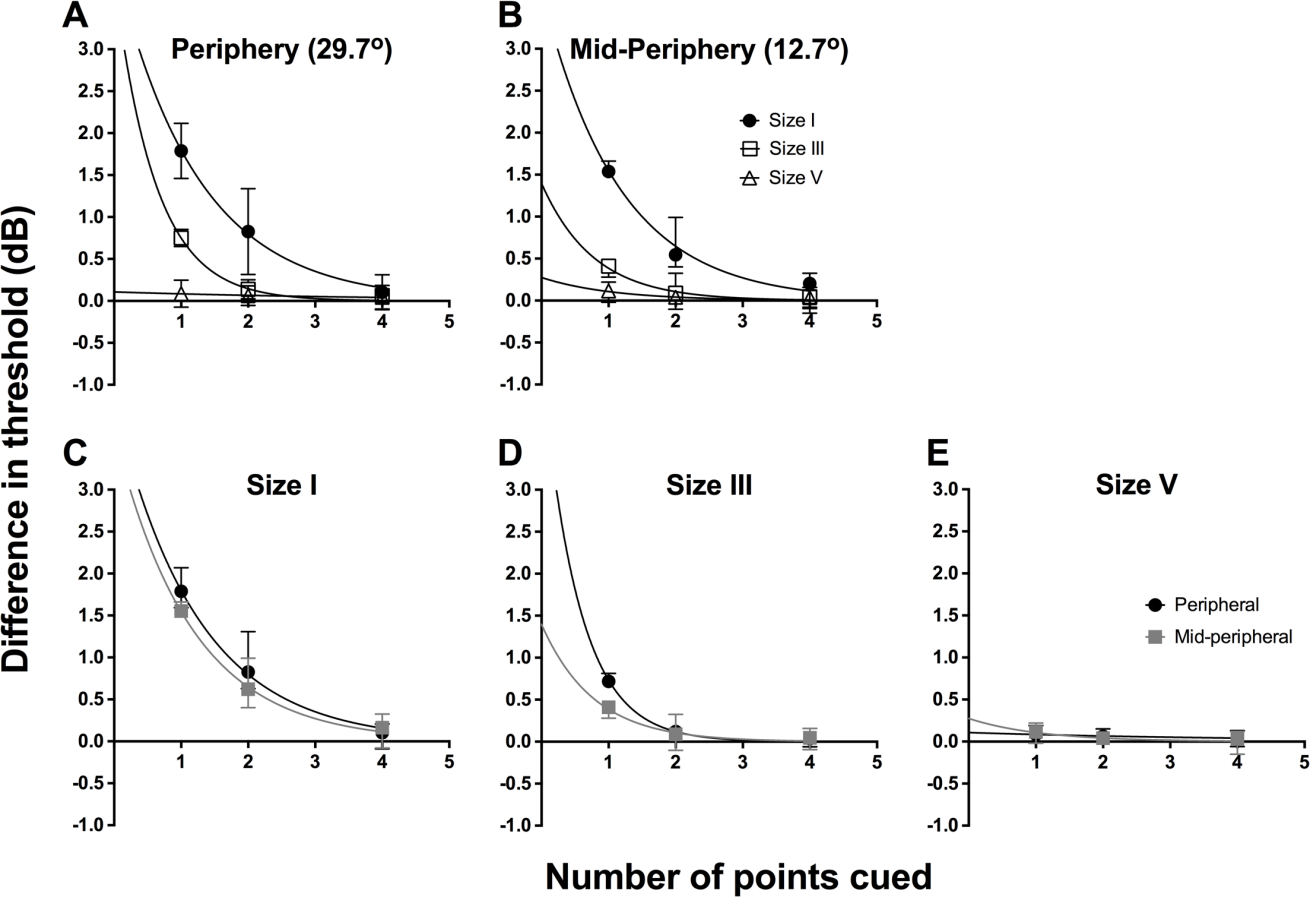
We plotted the absolute difference in threshold (in dB) between 8 points cued, which was considered the reference, and 1 point, 2 points and 4 points cued. Threshold was plotted as a function of number of points cued. Similar to Fig 3, the same data set was plotted in two ways: by stimulus location (A, B) and by stimulus size (C-E). The points here represent the average of the 4 observers, as these were absolute difference and thus could be combined. For all graphs, solid lines represent the best-fit one-phase nonlinear regression through those points, and error bars represent the range. The top row represents the functions sorted by peripheral (A) and mid-peripheral (B) stimulus location, and within each graph, we present size I (black circles), size III (open squares) and size V (open triangles) results separately. The horizontal dotted line (y = 0) indicates no difference between the number of points cued and the reference of 8 points cued. The resultant equations for the mid-peripheral (12.7°) conditions were: size I, y = 3.897e^-0.939x^; size III, y = 1.397e^-1.289x^; and size V, y = -0.027x + 0.131 (linear regression was utilised for size V). The resultant equations for the peripheral (29.7°) conditions were: size I, y = 4.061e^-0.815x^; size III, y = 4.487e^-1.814x^; and size V, y = -0.014x + 0.096 (linear regression was utilised for size V).

Table 1 shows the EC50 and slope results obtained from the sigmoidal nonlinear regression fits for the mid-peripheral (A) and peripheral (B) condition. Fig 4A–4E shows the difference plot of EC50 values between 1, 2 and 4 points cued, and 8 points cued, which we considered the reference due to the plateau found using the HFA, plotted in a similar fashion to that of Fig 4, again separated by eccentricity (Fig 4A and 4B) and stimulus size (Fig 4C–4E). These were combined between the observers as these represented the absolute difference between values. Repeated-measures two-way ANOVA was applied for the combined data set to examine the effect of number of points cued (factor 1, three levels) and location tested (factor 2, two levels). There was a significant effect for number of points cued (F(2,9) = 106.9, *p*<0.0001), increasing contrast sensitivity by approximately over 1.5 dB, but no effect due to eccentricity for size I stimuli (F(1,9) = 3.930, *p* = 0.079). The analysis for size III data confirmed a significant effect due to changing the number of points cued (F(2,9) = 41.87, *p*<0.0001) and when changing stimulus eccentricity (F(1,9) = 11.26, *p* = 0.0084). The magnitude of effect was smaller for size III than for size I. For size V stimuli, there was no significant difference due to stimulus size (F(2,9) = 1.098, *p* = 0.3744) or eccentric location (F(1,9) = 0.0091, *p* = 0.9262).

**Table 1:** The EC50 and slope values obtained from the nonlinear regression analysis for each condition in the mid-peripheral (A) and peripheral (B) conditions. The conditions are separated by stimulus size and the number of points cued, and are presented individually for each subject, due to the different contrast levels used. Hence, we also report the subject's age and gender (footnote). Using MoCS, we considered the EC50 point as the threshold, i.e. 50% frequency of seeing. Hence, we report EC50 as threshold values (in dB) in this table.

**A—Mid-periphery**													
											
**Stimulus size**		**Size I**				**Size III**				**Size V**			
**Number of points cued**		**n = 1**	**n = 2**	**n = 4**	**n = 8**	**n = 1**	**n = 2**	**n = 4**	**n = 8**	**n = 1**	**n = 2**	**n = 4**	**n = 8**
Subject 1	Threshold (dB)	1.16	2.37	2.59	2.82	-7.98	-7.65	-7.75	-7.70	-13.67	-13.67	-13.81	-13.69
	slope	0.82	0.53	0.41	0.47	0.74	0.61	0.62	0.61	4.86	4.42	3.96	5.08
Subject 2	Threshold (dB)	1.40	2.48	2.96	2.88	-6.65	-6.43	-6.36	-6.21	-14.08	-14.04	-14.03	-13.93
	slope	0.57	0.43	0.37	0.44	0.64	0.58	0.66	0.43	0.76	1.14	0.70	0.85
Subject 3	Threshold (dB)	5.60	6.56	7.02	7.20	-2.10	-2.00	-1.58	-1.67	-10.70	-10.58	-10.33	-10.48
	slope	0.45	0.31	0.38	0.35	0.50	0.54	0.44	0.43	0.60	0.79	0.65	0.47
Subject 4	Threshold (dB)	2.74	3.23	3.89	4.22	-5.72	-5.23	-5.37	-5.33	-13.77	-13.66	-13.66	-13.69
	slope	0.88	0.76	0.47	0.48	0.42	0.42	0.43	0.49	0.87	1.70	1.70	1.27
**B—Periphery**													
											
**Stimulus size**		**Size I**				**Size III**				**Size V**			
**Number of points cued**		**n = 1**	**n = 2**	**n = 4**	**n = 8**	**n = 1**	**n = 2**	**n = 4**	**n = 8**	**n = 1**	**n = 2**	**n = 4**	**n = 8**
Subject 1	Threshold (dB)	6.42	7.31	8.10	8.01	-4.28	-3.64	-3.63	-3.56	-10.11	-9.94	-10.06	-9.96
	slope	0.81	0.56	0.55	0.53	0.60	0.59	0.41	0.41	1.28	0.97	1.06	0.90
Subject 2	Threshold (dB)	5.20	6.25	6.72	6.88	-3.33	-2.66	-2.52	-2.51	-10.48	-10.61	-10.40	-10.46
	slope	0.69	0.62	0.55	0.57	0.69	0.43	0.36	0.35	1.34	1.18	1.25	1.01
Subject 3	Threshold (dB)	6.58	7.72	8.18	8.39	-1.41	-0.84	-0.62	-0.69	-7.67	-8.11	-7.85	-7.85
	slope	0.57	0.46	0.46	0.45	0.73	0.71	0.60	0.44	0.47	0.57	0.53	0.57
Subject 4	Threshold (dB)	4.97	5.73	6.91	7.04	-4.60	-4.01	-3.99	-3.92	-10.20	-10.10	-10.14	-10.01
	slope	0.41	0.47	0.29	0.31	0.44	0.38	0.50	0.37	0.48	0.50	0.49	0.51

Subject 1: 26 year-old male; Subject 2: 25 year-old female; Subject 3: 57 year-old male; Subject 4: 20 year-old female

We found a good fit with the exponential decay function, similar to the results from the HFA, for size I stimuli at 12.7° (R^2^ = 0.94) and 29.7° (R^2^ = 0.92) test locations, and size III stimuli at 29.7° (R^2^ = 0.98) locations. As expected, the fit was relatively poorer for size III at the 12.7° location (R^2^ = 0.60). As with the HFA result, linear regression appeared to fit the size V data better (R^2^ = 0.99 for 12.7°, and R^2^ = 0.93 for 29.7°).

**Fig 5:**
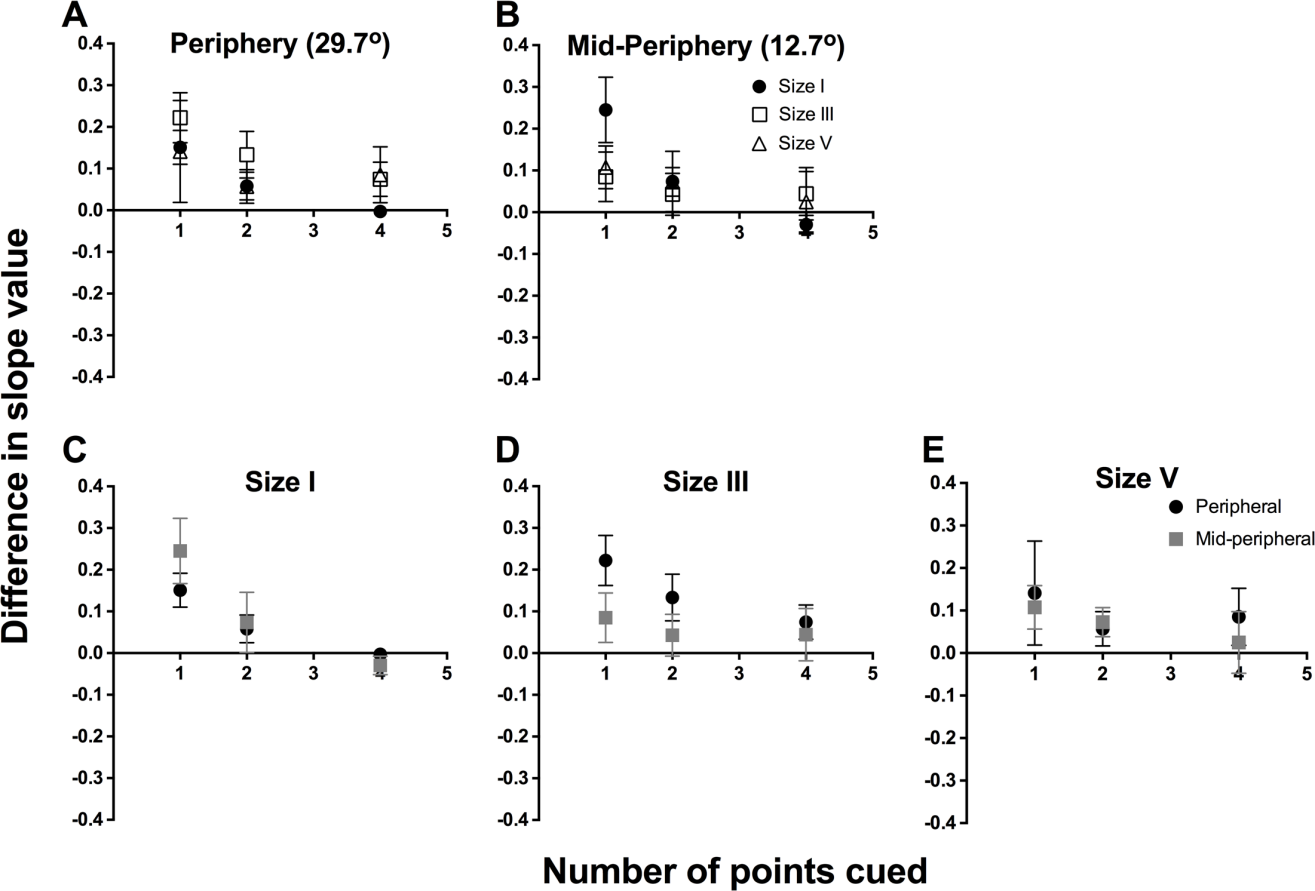
We plotted the difference in slope values between 8 points cued, which was considered the reference, and 1 point, 2 points and 4 points cued. Difference in slope was plotted as a function of number of points cued. Similar to Figs 3 and 4, the same data set was plotted in two ways: by stimulus location (A, B) and by stimulus size (C-E). The points here represent the average of the 4 observers, as these were absolute difference and thus could be combined. Error bars represent the SEM. The top row represents the functions sorted by peripheral (A) and mid-peripheral (B) stimulus location, and within each graph, we present size I (black circles), size III (open squares) and size V (open triangles) results separately.

In Fig 5, we show the difference plot of slope values between 1, 2 and 4 points cued, and 8 points cued, using the same method as above, separated by eccentricity (Fig 5A and 5B) and stimulus size (Fig 5C–5E), but combined across all four observers. A difference of 0 indicates no change in slope value; hence, the Figure depicts that when fewer points were cued, there was a trend towards a greater slope value, representing greater certainty. There was also a greater magnitude of difference seen at peripheral testing locations with cueing. Repeated measures two-way ANOVA found that number of points cued (F(2,9) = 6.543, *p* = 0.018) was a significant factor, but location was not (F(1,9) = 0.879, *p* = 0.373) for size I. For size III, number of points cued was not a significant factor (F(2,9) = 1.169, *p* = 0.354), but location was significant (F(1,9) = 5.162, *p* = 0.049). For size V, there was no significant effect of number of points cued (F(2,9) = 0.518, *p* = 0.613) or location (F(1,9) = 0.220, *p* = 0.651).

## Discussion

The pseudo-random nature of stimuli presentation in clinical SAP results in a measurement that attempts to quantify the contrast sensitivity threshold at multiple spatial locations in the visual field, but in doing so, introduces spatial uncertainty as a parameter affecting that measurement. Our results demonstrate that verbally cueing the location and number of points for size I, or equivalent, stimuli increased contrast sensitivity, but did not affect the results for size V stimuli. In the case of size III stimuli, the location of the presentation also affected the magnitude of improvement in performance. Taken together, these results are consistent with previous studies using similar [22] or slightly different paradigms [14, 15, 19, 39], which have shown that cueing certain stimulus properties facilitates improvement in detection ability. The effect of cueing was found to diminish with increasing number of elements cued, eventually returning to baseline performance following an exponential decay [40], consistent with previous studies on working memory showing that observers can accurately recall the visual details of three or four elements [41, 42], and allocation of attention to multiple spatial locations [39] and set sizes [43]. The lack of effect with size V stimuli is consistent with previous suggestions that larger stimuli are less subject to the effects of attention and variability [27, 44], as stimulus size has been described to be a guiding attribute for visual attention [45], visual search tasks [46] and task difficulty [19, 43].

We found that cueing made a bigger difference in the peripheral region compared to mid-peripheral region when using a size III stimulus. One possible reason for this is the rate of change sensitivity across the visual field. Sensitivities across the visual field have been found to differ according to stimulus size, which is commonly depicted in cross-section form resembling a ‘hill of vision’ [11, 23, 25, 47]. For example, a size I stimulus displays a relatively steep ‘hill’, signifying a greater rate of change of sensitivity with increasing eccentricity. One explanation for constant effect of cueing at peripheral and mid-peripheral test locations for size I may be due to a peak in the level of spatial uncertainty at both eccentricities used in the present experiment. In comparison, a size V stimulus undergoes minimal change; previous studies have shown a flatter ‘hill’ [11, 22, 25], which may be related to the lesser uncertainty seen in our results. A size III stimulus, however, has a relatively flat ‘hill’ up to the mid-periphery, then displays a steeper change in the periphery, and this may therefore manifest as differences in the magnitude of increase in sensitivity at different eccentricities.

The two predominant theories on the mechanism of cueing affecting sensitivity at near-threshold levels are signal enhancement and uncertainty reduction [14, 48–50]. For example, uncertainty reduction improves sensitivity by reducing the effects of noise or distractor stimuli through foreknowledge of the target location [16, 18, 20]. The results of this study may be explained by uncertainty reduction as the paradigm we have used reduced spatial uncertainty through attentional cueing, and because we presented targets at multiple possible locations [51]. This appears consistent with Pelli’s [16] models on contrast detection and uncertainty factors, as extrinsic factors in the present study have been reduced with cueing and intrinsic factors through the use of experienced observers. The results of the laboratory-based testing also appear to be consistent with the work of Lasley and Cohn [52], who showed a positive acceleration in the psychometric function when spatial uncertainty is introduced; this is seen in the gradual slope in the uncertain conditions in our results. Our results can be also explained by postulations that visual attention is analogous to the eye having its own spotlight [53, 54] or telescopic zoom lens [55–57], in that attentional focus is localised to a small area. Increased division of attention then leads to an exponential decrease in sensitivity [55].

Our results have a number of possible clinical implications. A 2 dB change is a substantial change in threshold static perimetric testing. Artes et al. [31] have shown that at an average sensitivity of approximately 30 dB there is an estimated test-retest variability of 2 dB using SITA Standard paradigm on the HFA. An error of 2 dB due to attentional cueing therefore approximately represents the top 5 percentile of test-retest variability. Furthermore, the algorithm used in the full threshold paradigm in the HFA is a 4–2 staircase procedure, where threshold is taken as the last 2 dB reversal. Our results could not be explained by test-retest variability alone–or practice effects, as our subjects were highly experienced observers and perimetric subjects, as the standard deviation of our HFA data spanned approximately 0.25 dB–substantially below the 2 dB test-retest variability; therefore, we suggest that spatial uncertainty contributes to variability in addition to that of test-retest variability.

We hypothesise that there is unlikely an effect of cueing due to pseudo-random order of SAP, as, after the first four seeding points, there are 16 possible “surrounding” points that can be tested: well above the number of elements where we found a plateau effect. As testing progresses, the number of possible locations for stimuli to appear increases, thus further increasing spatial uncertainty. In particular, we found that cueing effects for size III stimuli are affected by test location: peripheral eccentricities appeared to be subject to greater spatial uncertainty, and may therefore result in greater variability in thresholds obtained in the peripheral visual field [35].

Practically, retesting individual points in SAP (for example, in the commercially available Medmont Perimeter) in which a Goldmann size III is utilised, our results suggest that at least 8 points should be retested to minimise the potential effect of attentional cueing. In addition, custom visual field test patterns should also utilize at least 8 points for the same reason. However, more observers drawn from the general population need to be tested to determine the true clinical implications of attentional cueing.

The present study employed a small number of experienced observers with normal vision that, while sufficient to confirm the effect of spatial uncertainty on contrast detection thresholds, is insufficient to make generalizations to a larger, normal population. Future studies might also examine a greater number of normal observers inexperienced at the psychophysical task to extrapolate these results to the general population, and to determine the effects, if any, of practice on the cueing effect, as training may affect certainty.

The effects of attentional cueing may also differ in those with visual field deficits. For example, previous studies have previously shown that variability may be increased in defective regions of the visual field in patients with disease like glaucoma [26, 58, 59]. The FOS curves in regions of field loss have been shown to be different in comparison to equivalent regions tested in normal subjects: there is an increase in threshold, as well as a relative flattening of the FOS curve. The flattening of FOS curves in glaucoma is thought to indicate increased variability [26], most likely due to underlying progressive cellular loss, whereas size V stimuli offset this uncertainty in glaucoma patients by reducing the signal-to-noise ratio [27]. Future studies could examine whether attentional cueing could alter the variability in thresholds and shape of the FOS curve in patients with glaucoma or other diseases affecting the visual field, and these results may differ with varying depth of field loss.

More rigorous psychophysical testing at locations that are finely-tuned to directly match the HFA test locations, could determine whether differences in cueing effect exist, and whether this effect changes in a location-specific manner, such as points that are more adjacent to each other. Although a visual cue is unavailable using clinical perimetry instruments, a future experiment comparing the effects of verbal and visual cues would be informative, as these have been shown to have different characteristics such as span and ageing effects [60].

## Supporting Information

S1 Fig Individual data for the difference in threshold between cued and uncued conditions (dB) as a function of the number of points cued for peripheral test locations. Error bars represent 1 SEM. Size I (black circles), size III (open squares) and size V (open triangles) results are presented separately. Solid lines represent the best-fit one-phase nonlinear regression through those points.
(TIF)

S2 Fig Individual data for the difference in threshold between cued and uncued conditions (dB) as a function of the number of points cued for mid-peripheral test locations. Error bars represent 1 SEM. Size I (black circles), size III (open squares) and size V (open triangles) results are presented separately. Solid lines represent the best-fit one-phase nonlinear regression through those points.
(TIF)

S3 Fig The frequency-of-seeing (FOS) curves for subject 1, which plot proportion seen as a function of log contrast (in dB, Δ*L*/*L*). The top row (A-C) consists of the curves for the mid-peripheral condition, and the bottom row (D-F) consists of the curves for the peripheral condition. The left, middle and right curves are the results for size I, size III and size V respectively. The results for 1 point, 2 points, 4 points and 8 points cued are represented by the colours black, blue, red and yellow respectively. These results represent the averaged result of 20 trials for each contrast level.
(TIF)

S4 Fig The FOS curves for subject 2, plotted as per the method in S3 Fig. (TIF)

S5 Fig The FOS curves for subject 3, plotted as per the method in S3 Fig. (TIF)

S6 Fig The FOS curves for subject 4, plotted as per the method in S3 Fig. (TIF)

S1 Table Weber contrast levels used for each subject under each size and location condition. (TIF)
