# Supplementary figures and images for: The Effect of Attentional Cueing and Spatial Uncertainty in Visual Field Testing
Source: PLoS One. 2016 Mar 3;11(3):e0150922. doi: 10.1371/journal.pone.0150922 (PMC4777401; doi:10.1371/journal.pone.0150922)

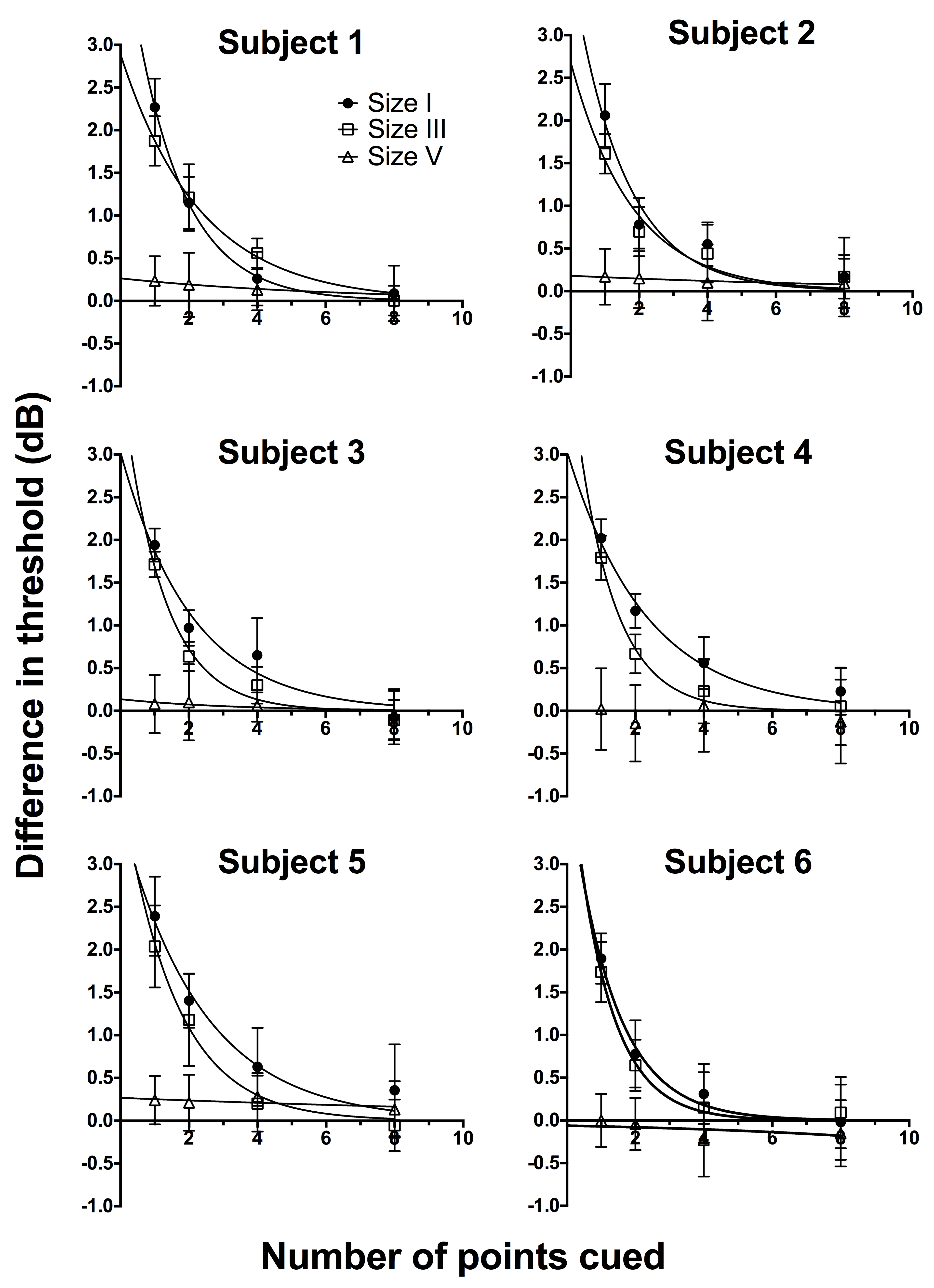

Supplement: S1 Fig — Error bars represent 1 SEM. Size I (black circles), size III (open squares) and size V (open triangles) results are presented separately. Solid lines represent the best-fit one-phase nonlinear regression through those points. (TIF) [file pone.0150922.s001.tif]

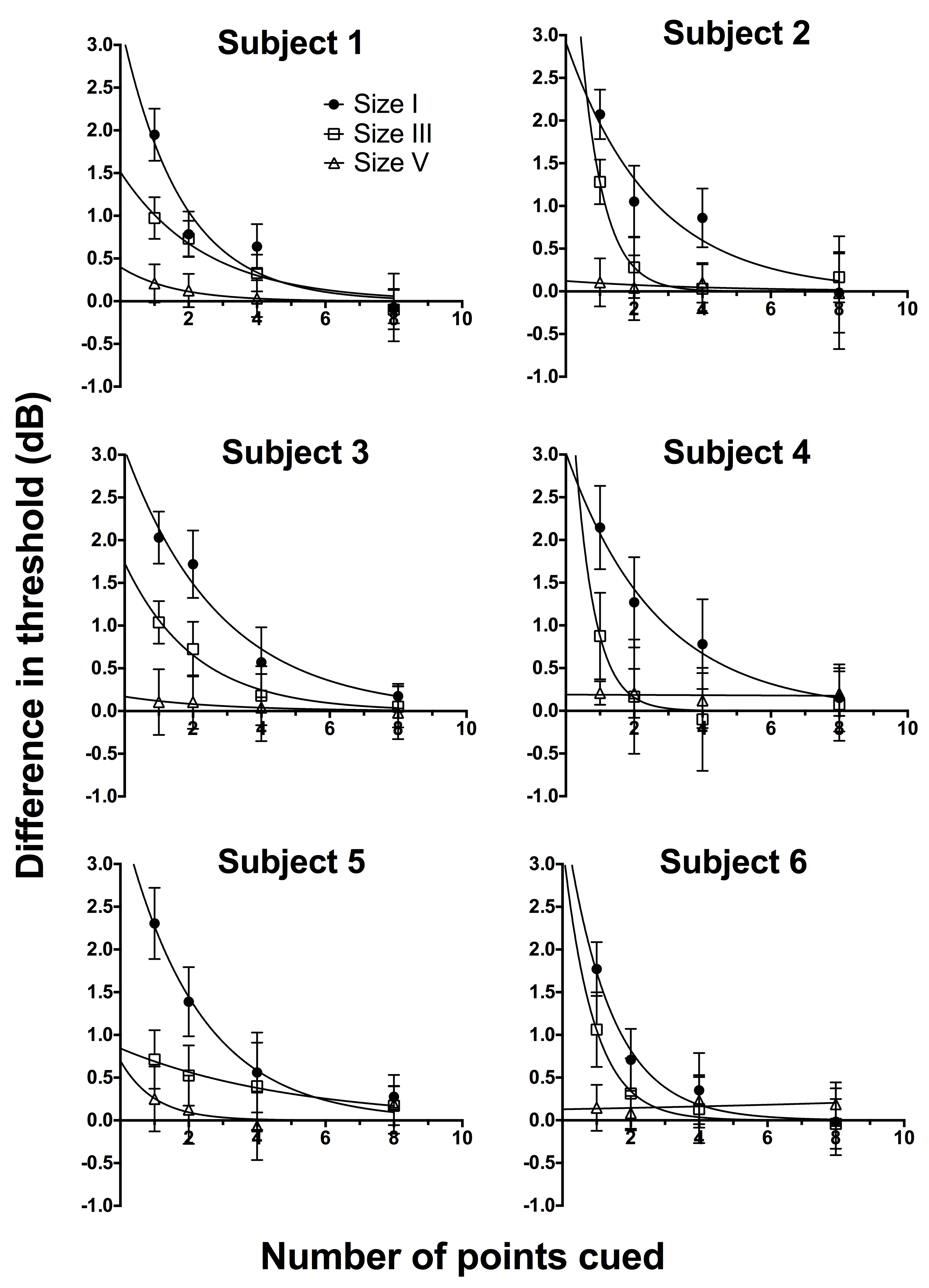

Supplement: S2 Fig — Error bars represent 1 SEM. Size I (black circles), size III (open squares) and size V (open triangles) results are presented separately. Solid lines represent the best-fit one-phase nonlinear regression through those points. (TIF) [file pone.0150922.s002.tif]

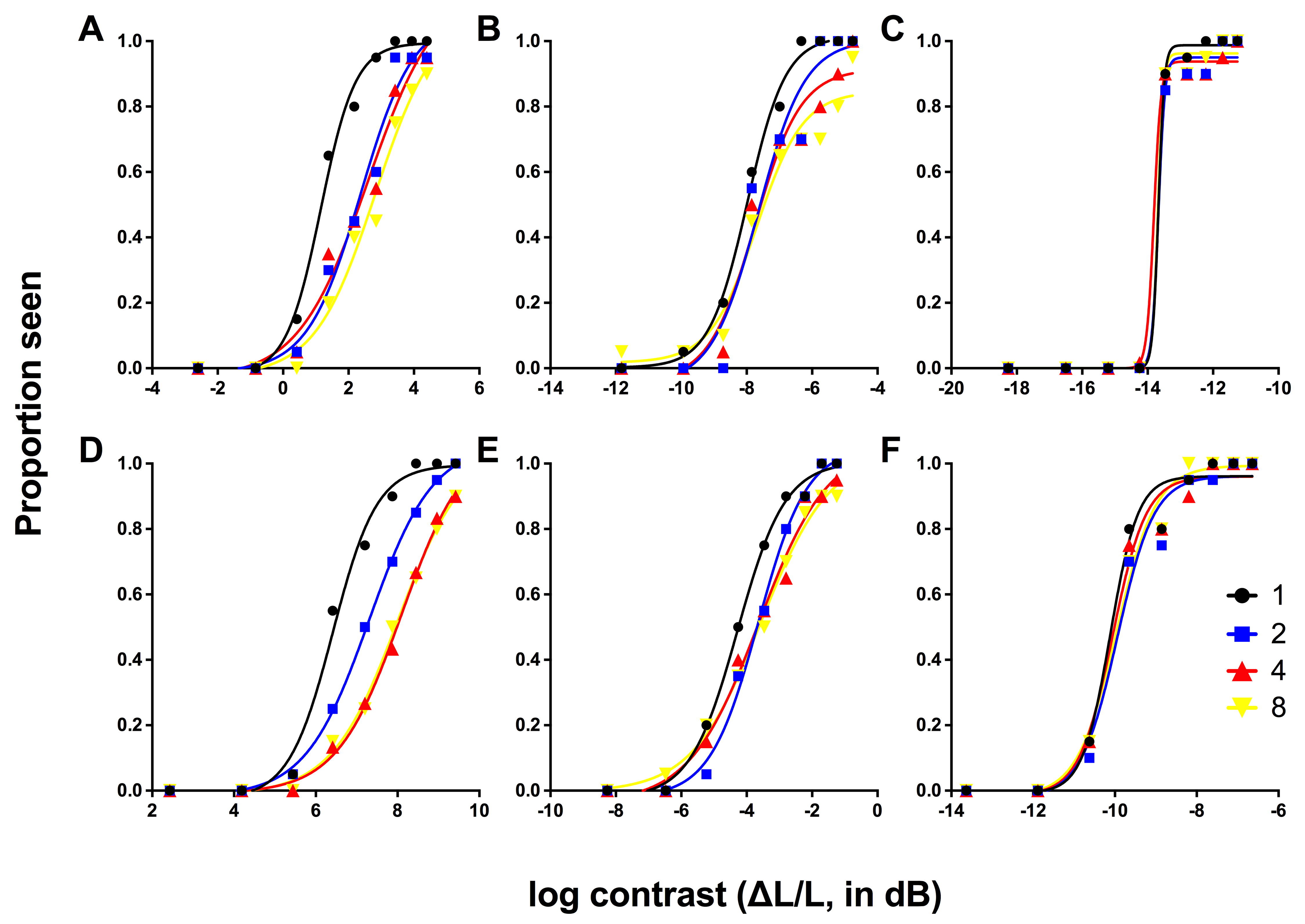

Supplement: S3 Fig — The top row (A-C) consists of the curves for the mid-peripheral condition, and the bottom row (D-F) consists of the curves for the peripheral condition. The left, middle and right curves are the results for size I, size III and size V respectively. The results for 1 point, 2 points, 4 points and 8 points cued are represented by the colours black, blue, red and yellow respectively. These results represent the averaged result of 20 trials for each contrast level. (TIF) [file pone.0150922.s003.tif]

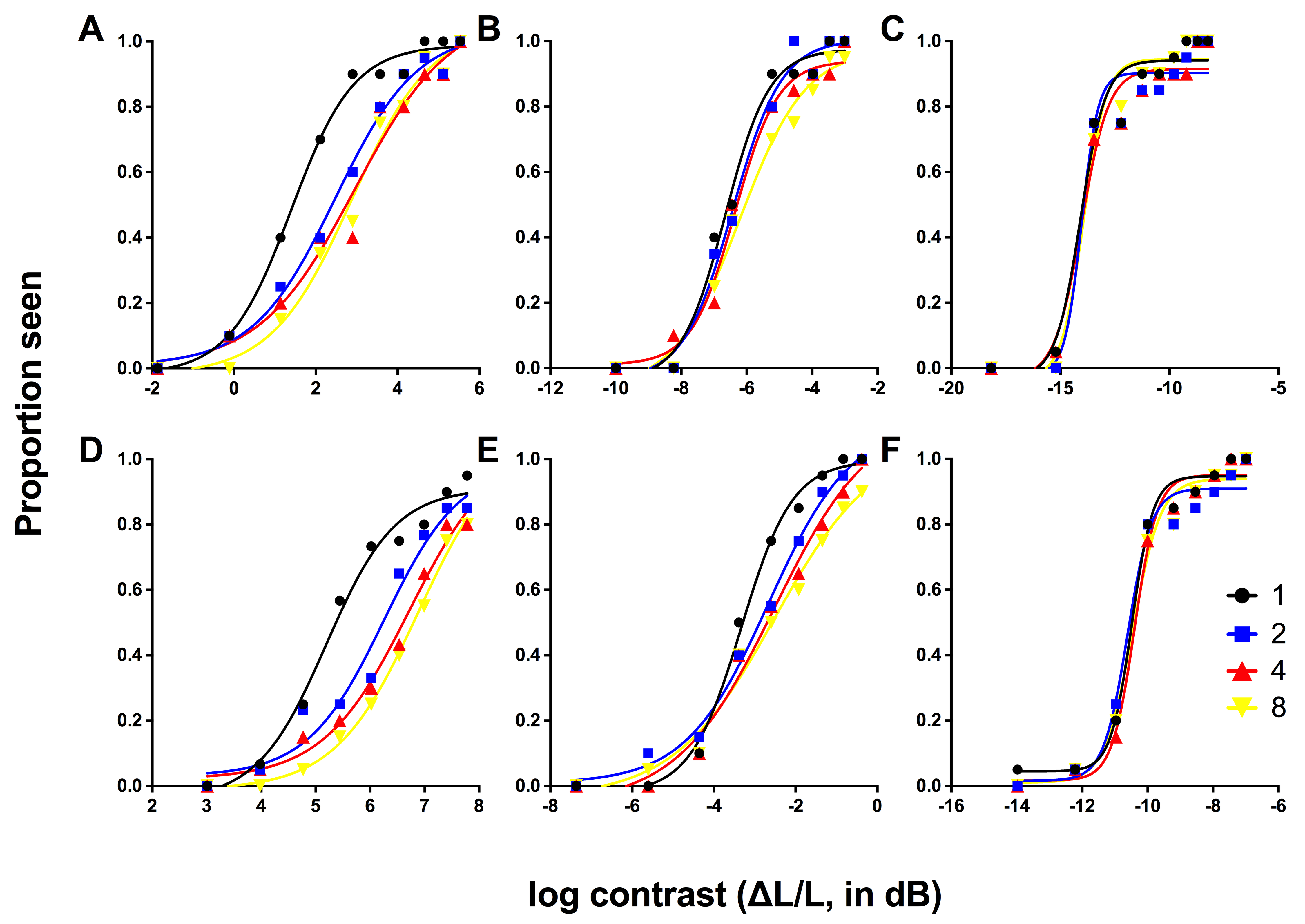

Supplement: S4 Fig — (TIF) [file pone.0150922.s004.tif]

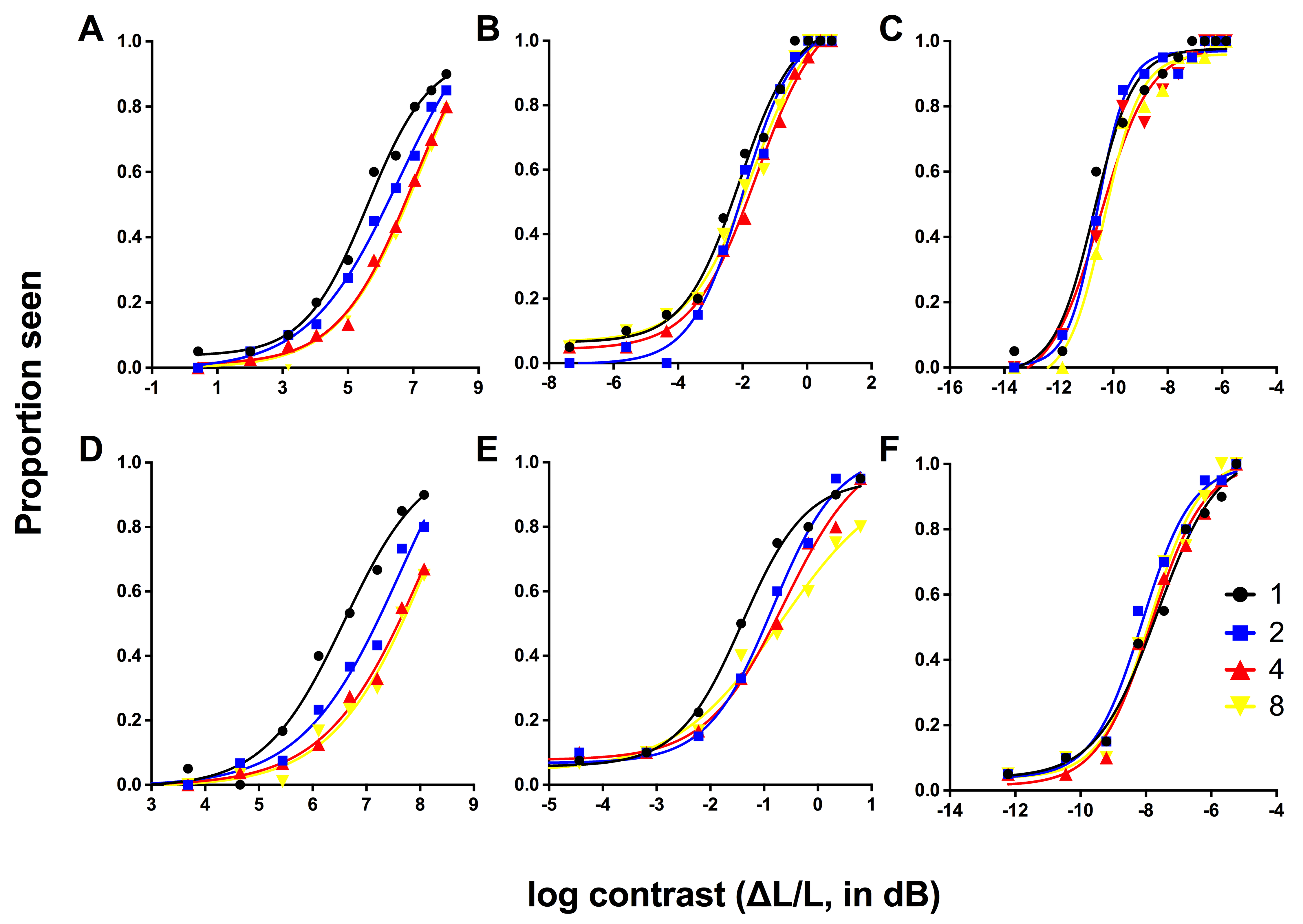

Supplement: S5 Fig — (TIF) [file pone.0150922.s005.tif]

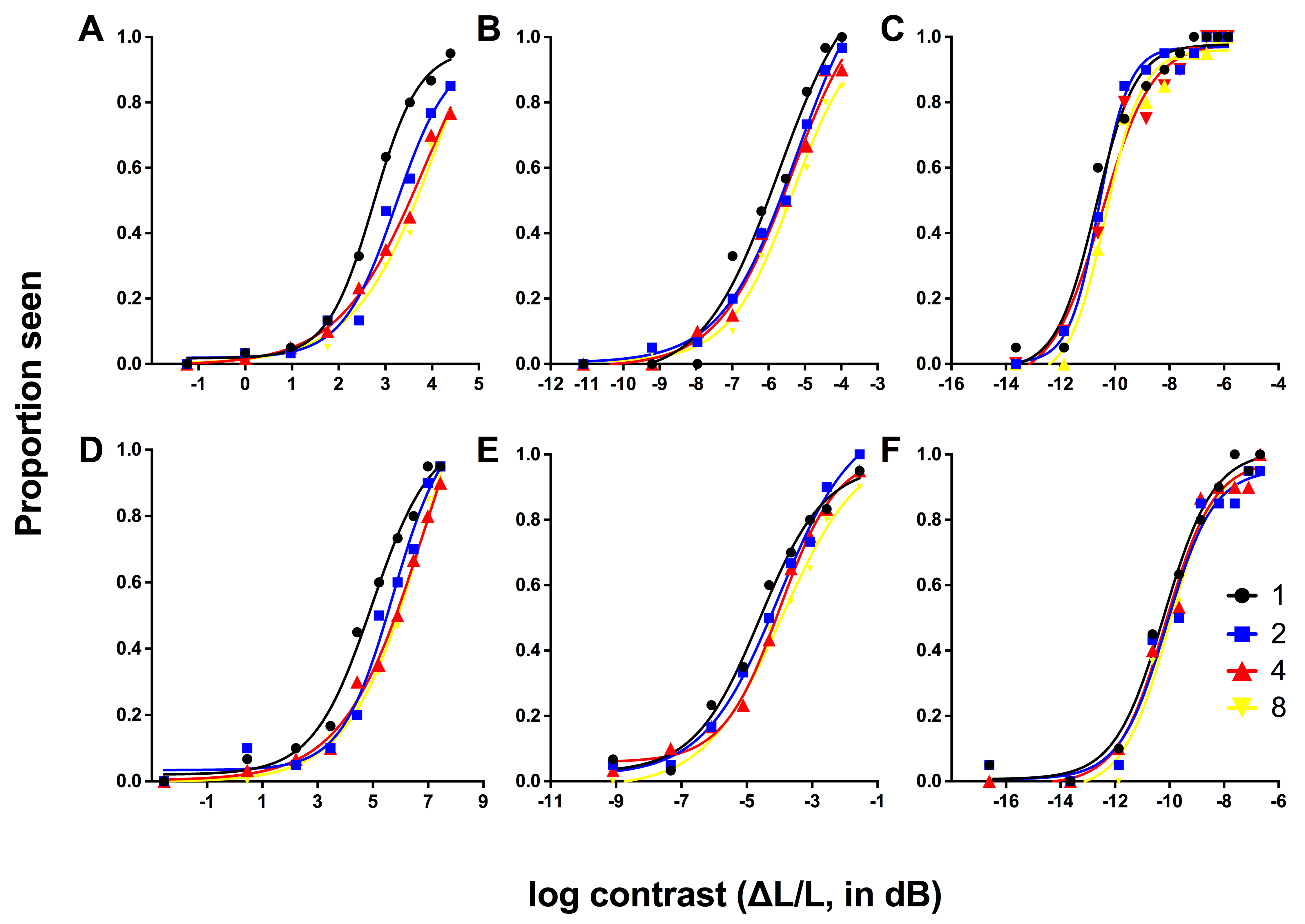

Supplement: S6 Fig — (TIF) [file pone.0150922.s006.tif]

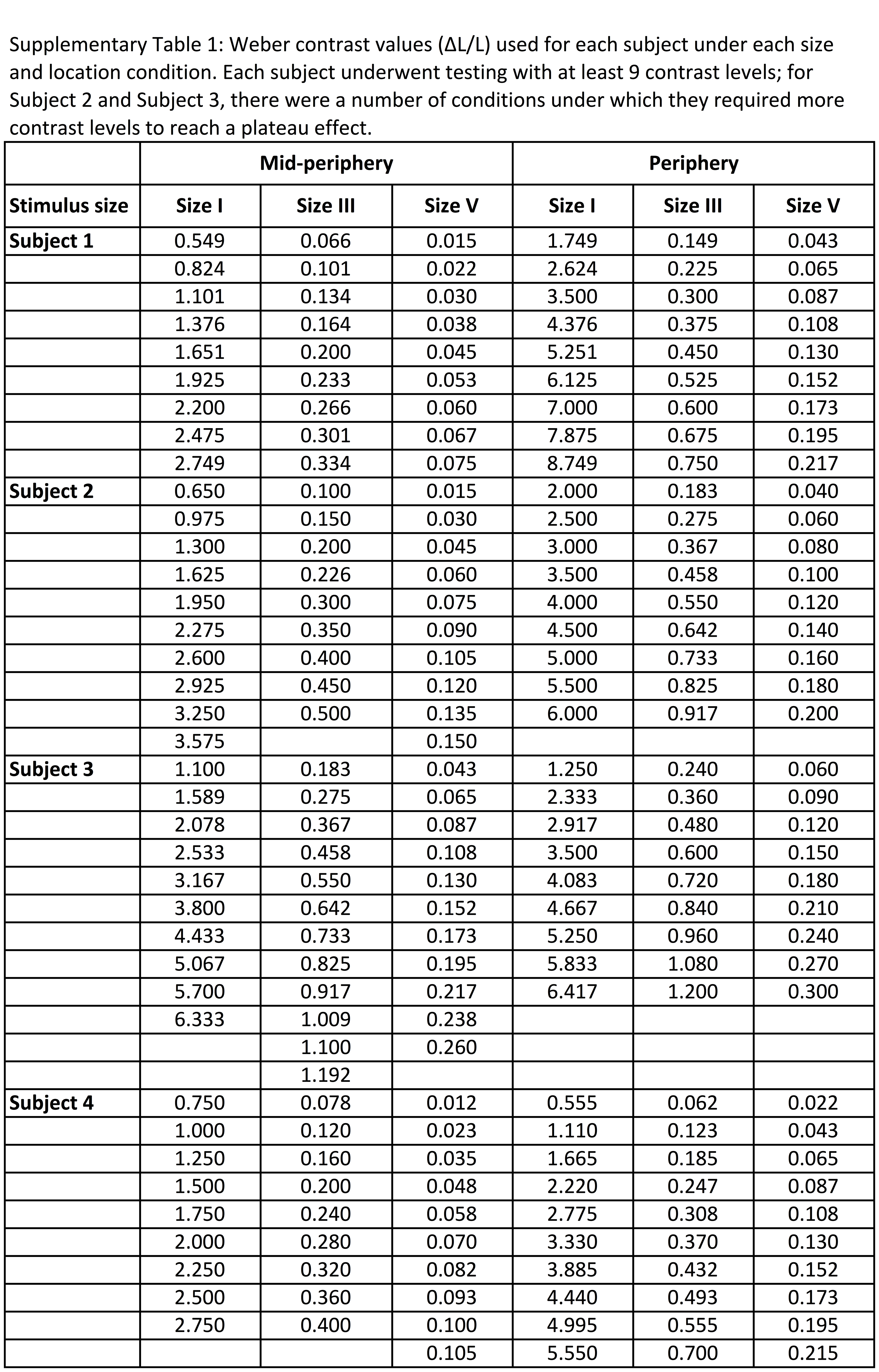

Supplement: S1 Table — (TIF) [file pone.0150922.s007.tif]
